# Acceptance of COVID-19 vaccine booster dose among the people of Bangladesh: A cross-sectional study

**DOI:** 10.1016/j.heliyon.2023.e22215

**Published:** 2023-11-11

**Authors:** Debendra Nath Roy, Shaheb Ali, Ashish Kumar Sarker, Ekramul Islam, Md. Shah Azam

**Affiliations:** a Department of Pharmacy, Jashore University of Science and Technology, Jashore-7408, Bangladesh; b Institute of Education and Research, University of Rajshahi, Rajshahi-6205, Bangladesh; c Department of Pharmacy, Pabna University of Science and Technology, Pabna, Bangladesh; d School of Science, Western Sydney University, NSW-2560, Australia; e Department of Pharmacy, University of Rajshahi, Rajshahi-6205, Bangladesh; f Department of Marketing, University of Rajshahi, Rajshahi-6205, Bangladesh; g Office of the Vice Chancellor, Rabindra University, Bangladesh

**Keywords:** COVID-19, Vaccine booster, Acceptance, People, Bangladesh

## Abstract

Vaccine booster dose (VBD) provides a potential therapeutic alliance in preventing breakthrough infection and new variant's arrival while preserving long-lasting host immunity. This study aimed to analyze COVID-19 VBD willingness and identified the key determinants of VBD acceptance among the general people of Bangladesh. This survey-based study applied a quantitative research paradigm. A validated, anonymous, and multi-item questionnaire was adopted through a theoretical review of pertinent literature on the topic. Data were collected between August 2022─October 2022, and sampling was done randomly. A total of 704 individuals were invited via face-to-face interview approach; however, 13.8 % of them declined to give consent, which resulted in the participation of 607 potential respondents. The main outcome measure was COVID-19 VBD acceptance willingness. Binary logistic regression analysis was conducted to rationalize the study's objectives. The pooled COVID-19 booster vaccine acceptance rate was 70.0 % (95 % confidence Interval [CI]: 67─73) among Bangladeshi people. An analysis of binary logistic regression revealed that, out of 14 potential factors, “efficacy”, “repeated immunity”, ”communication”, and “trust” showed highly significant positive association (adjusted odds ratio [aOR ] = 2.151 95 % CI: 1.391─ 3.508, aOR = 2.033 95 % CI: 1.299─ 3.181, and aOR = 2.552 95 % CI: 1.557─4.183 respectively, *p<0.01*), and “equal safety”, “risk-benefit ratio” and “community protection” had significant positive association (aOR = 1.739 95 % CI: 1.070─2.825, aOR = 1.712 95 % CI: 1.116─2.627, and aOR = 1.628 95 % CI: 1.395─0.998, *p<0.05)* with VBD acceptance*.* However, post-vaccination “side effects” showed significant negative (aOR = 0.393 95 % CI: 0.237─0.674, *p<0.01*) associations with VBD acceptance. The odds of accepting the COVID-19 vaccine booster was found 1.26, and it was found insignificant (*p>0.05)* in the Chi-squared test. Bangladeshi people expressed a moderately high level response to COVID-19 VBD acceptance. A positive attitude towards the COVID-19 VBD is an outcome of this study, regardless of the circumstances, as far as safety, efficacy, perceived health benefits, communication, trust, and community resistance are concerned. Post-vaccination side effects fear was the primary reason for booster dose skepticism as well as a barrier to administering booster shots. The confidence in COVID-19 VBD will be boosted when mass people are effectively communicated and vaccine's data become more available publicly.

## Highlights

• In order to limit the COVID-19 pandemic, the therapeutic alliance through booster vaccination was a fundamental health intervention modality.
• COVID-19 booster vaccine acceptance rate was 70.0 % among Bangladeshi people, and several key antecedents were associated with vaccine booster dose decisions.
• Post-vaccination side effects fear is the reason for booster dose skepticism as well as a barrier to administering booster shots.
• A collaborative approach and public compliance are required for country-wide COVID-19 vaccination drive to produce sustainable host immunity.

## 1 Introduction

Over three years, the global outbreak of novel coronavirus disease (COVID-19) has become one of the grea**t**est threats to public health and is considered the largest health risk and humanitarian tragedy over time that have seen yet. Vaccines are evaluated as the safest option for developing herd immunity in individuals with flu pandemics [1,2]. Most vaccine candidates targeted an endemic to limit viral loads and thereby arrest quick transmission. Vaccines are rare in emergencies where the prospective health benefits are greater than uncertain risks [3]. While disclosing the multiple genome sequences of novel coronavirus, over 100 vaccine candidates has been included into the platform [4]. The vaccines effectively crossed pre-clinical and randomized clinical trials against COVOD-19 [5]. Since late 2020, the availability of COVID-19 vaccines opened up the option of preventing the current pandemic fast. To control the infectivity, 60–80 % of a population must establish herd immunity, depending on the clinical effectiveness of the vaccine being administered [6]^.^ [7]. However, vaccine accessibility and costs, perceived side effects, and public acceptability were essential determinants of successful vaccination drive [8].

While newly produced vaccinations were intended to lower the risk and the spread of COVID-19, recent reports have increasingly shown a reduction in herd immunity [9,10]. Even though massive vaccinations were the most effective approach in limiting corona infections, consecutive waves of infection have occurred in several countries [11]. These waves could be the result of changes in various corona virus strains, which could contribute to partial vaccine resistance and the long-term decrease of acquired immunity to vaccines. These waves may be the consequence of mutations in several strains of the corona virus, which may assist in partial vaccine resistance and the gradual decline of vaccine-developed immunity over time [[12], [13], [14]]. Thus, the World Health Organization (WHO) and global scientists decided to boost the third dose of vaccine against COVID-19. Surprisingly, booster doses showed a significant recovery of antibody levels, especially in elderly and immune-compromised individuals with limited response to standard vaccination courses and a high risk of relapse [15]. Scientific data reported that, booster vaccines administration has increased defense mechanism against COVID-19 infection and severe sickness of 11.3-fold and 19.5-fold, respectively [16].

Despite booster vaccine doses plays a leading role in limiting the emergence of new variant in the global health community [17]; however, hesitancy to receive a booster dose has been recognized as a barrier to boost a third vaccine dose. Vaccine hesitancy was predominant among various sub-population groups for primer COVID-19 vaccination drive [[18], [19], [20], [21]] and now for booster vaccine shot [[22], [23], [24]]. Global disparities in COVID-19 VBD acceptance were well documented across the countries, particularly in under-developed countries, and peoples are still reluctant to administer COVID-19 VBD [25,26]. Although few researchers reported relatively low levels of COVID-19 VBD apprehension in developed [[27], [28], [29]] and developing countries [[30], [31], [32]], a significant variability in responding COVID-19 VBD was found across different societies within the country [33]. Worldwide, several multi-dimensional antecedents were associated with COVID-19 VBD receptivity including socio-economic (e.g., employment, education level, and income status), socio-demographic attributes (e.g., marital status, age, gender, and ethnicity), place of residency (e.g., urban, semi-urban/rural), vaccine-specific concerns (equal safety, efficacy, effectiveness and post-vaccination side effects) socio-psychological (e.g., anti-vaccination sentiment, perceived risk of contagion, self-efficacy, sense of control, and academic attainment), financial (e.g., income source, living status), environmental (e.g., information sufficiency, trust, communication), experiential (e.g., disease exposure, social influences, previous vaccination experiences, and a history of clinical studies) influenced VBD decision [[34], [35], [36], [37], [38]].

Since April 2022, Bangladesh has started to administer COVID-19 booster vaccine shot in the wake of repeated exposure, the emergence of unseen mutant, and a wane in resilient immunity. Primarily, the policymakers decided to deliver booster doses among the front liners and the elderly as a priority group. The government now plans to deliver booster shoot among the general people over 18 years aged amidst to stop new variant transmission. Whilst to date, several observational studies focused on primer COVID-19 vaccination consequences among various sub-population groups in Bangladesh [[39], [40], [41], [42], [43]]. However, there had been no assessment of public response and readiness to receive COVID-19 vaccine booster doses in Bangladesh. Therefore, the purpose of this study was to investigate the acceptance of COVID-19 vaccine boosters and to explore the multi-dimensional factors that may influence public acceptance of booster doses in Bangladesh.

## 2 Methods

### 2.1 Study design

This cross-sectional study employed a quantitative research design. An anonymous, multi-items, closed-ended bilingual questionnaire was developed to fine tune the study's key objectives. Data were collected randomly in a face-to-face interview approach from August 2022 to October 2022. The methods used were all in accordance with the relevant guidelines and regulations for involving human subjects. The detailed research protocol was reviewed by the **Ethical Review Committee” (ERC) before the study began** and approved the study as exempt. We deposited step-by-step descriptions of the research protocols on protocols.io (DOI: dx.doi.org/10.17504/protocols.io.j8nlkw88wl5r/v1); thus, the reproducibility of the study method was established. No external funding was available**.**

### 2.2 Setting and participants

This study recruited encounters with the general people of Bangladesh aged 18 and over. Required data were collected across the eight divisional parts in Bangladesh for ensuring the reproducibility and generalizability of the findings. Eligible participants were requested to provide an informed consent and not rewarded in kind or financially. *Participation was completely voluntary and the participants were not harmed by research paradigm.* A total of 704 individuals were interviewed for this quantitative data assembly, while 13.8 % of them declined to provide informed consent.

### 2.3 Participant recruitment criteria

The inclusion criteria of participants were the followings: (i) having a clear understanding and agreeing with the study objectives, (ii) self willingness to provide anonymous data on COVID-19 booster vaccination, (iii) age 18 years and above, and (iv) received 1st dose and/or 2nd dose of COVID-19 vaccine.

The exclusion criteria were: (i) age below 18 years, ii) not agreed to give informed consent, and (iii) not vaccinated by a COVID-19 primer dose.

### 2.4 Measures and survey instrument development

Analysis of recent and relevant systematic reviews has provided a theoretical underpinning for the concept of COVID-19 vaccine acceptance and hesitancy [18,34,44]. Key items of the questionnaire were adopted from a theoretical analysis of the recent studies conducted on COVID-19 VBD acceptance across the countries [[45], [46], [47], [48]]. Moreover, in-field consultation was conducted in constructing and rationalizing the relevant questionnaire items. We developed a validated close-ended questionnaire composed of twenty-seven items. The socio-demographic profiles of the participant were scaled in nominal and the ratio scale was used for multiple type measurements. Dichotomous binary questionnaires (yes and no option) were nominally adopted to construct self-explanatory outcome and predictor variables of interest. For instance, we measured the outcome response asking a single item: “I would like to accept COVID-19 vaccine booster dose anytime (outcome response)” and multiple predictor variables were measured using following items: “I confirm that booster doses are similarly safe as were regular two doses”; “I think the vaccine booster dose is very efficacious”; “I am worried about the post-vaccination side effects of vaccine booster dose”; “Vaccine booster doses may provide repeated immunity to the body”; “I am well-communicated about booster vaccine dose”; “I have trust on booster vaccination”; “Therapeutic benefit of booster doses overweigh the perceived health risk”; “I have adequate information on vaccine booster doses”; “Booster vaccination is essential to protect my community people”; “I do not need booster doses because I strictly follow the community protective measures”; “Booster vaccine doses should be mandated for people”; “Booster vaccine doses prevent the arrival of new corona variant”; “Booster vaccine provides long-lasting immune responses”; “Receiving a vaccine dose is very usual to me”.

The questionnaire items were originally developed in Bengali (native) language and translated into English. The average time of 20 min was spanned for completing the questionnaire items. The survey distribution used in-person data collection because this mode of distribution reached the target population more effectively, got maximum and fast response rate, and offered better quality data. We recruited eighteen surveyors across the divisional areas of the country to support in-person data collection and survey distribution process. The survey instrument analyzed the followings: (1) socio-demographic and socio-economic overview of the encounters, (2) willingness to uptake COVID-19 VBD, and (3) the potential determinants of COVID-19 VBD acceptance and hesitancy.

### 2.5 Validity and reliability of the measurements

Literatures were analyzed and reviewed on the topic for ensuring the validity of the study measurements. Items of the questionnaire were content and face-validated using a panel of public health expert; thus relevance and clarity of constructs were established. Pilot testing (n = 20) was undertaken to validate the legitimacy of the survey instrument and the results of the piloted data were precluded from the final analysis. The reliability of the dichotomous questionnaire in the measurement was confirmed by examining the value of Cronbach's alpha (α) and Kuder-Richardson (KR-20). The value of Cronbach's alpha (α) and KR-20 was estimated 0.84 and 0.81, respectively, which is assumed to be a good scale for providing the internal consistency of the measurement.

### 2.6 Outcome variable

The response/outcome variable of the study assessed the acceptance of the COVID-19 vaccine booster dose. The outcome question was, “Would you like to receive COVID-19 booster vaccine dose anytime”? In this question, participants could reply whether they had booster acceptance intent, not sure, and not willing to uptake. Using a binary variable, acceptance intent was categorized as yes = 1, while the other two responses were transformed into otherwise = 0.

### 2.7 Independent variables

The independent variables were adopted from recent studies on COVID-19 booster vaccination consequences performed in regional and global contexts, particularly observations in the South Asian regions. These variables were grouped as; contextual factors (socio-demographic and socio-economic profiles of the participants), socio-psychological determinants, and vaccine-specific predictors. For a binary analysis, we examined the effect of numerous potential factors on outcome variables (booster vaccine dose acceptance) dichotomized into 1 = yes response and 0 = unsure and no responses.

### 2.8 Study size

We used SurveyMonkey equation for determining the least required sample size with a confidence interval of 95 % (z score of 1.96) and a 5 % margin of error.

(1) $$\text{sample}\;\text{size}=\frac{\frac{z^{2}\times p\left(1-p\right)}{e^{2}}}{\frac{1+\left(z^{2}\times p\left(1-p\right)\right)}{e^{2}N}}$$

Where N = population size, p = sample probability, e = margin of error (percentage in decimal form), and z = z-score.

In general, binominal regression analysis with large sample sizes that characterized the parameters was advised for observation studies with a minimum of 500 data. A similar equation was also considered (n = 100 + 50i), where i stand for the number of independent variables [49,50]. As a pilot study, we pre-tested the data samples (n = 20) to evaluate the clarity of the instruments and to determine the average response time.

### 2.9 Data management and analysis

Auditing, coding and sorting of the collected questionnaire was checked manually every day to ensure the completeness of the survey tool. After checking the completeness of the data, the data were entered in Microsoft Excel 10 and then exported to Statistical Package for the Social Science (SPSS) software. IBM-SPSS version 25 (RRID: SCR_016479). Descriptive analysis was done for both dependent and independent variables and presented in terms of frequency, mean, percentage and text. In order to identify potential confounding variables, binary logistic regression analysis was performed, and variables with a *p*-value <0.05 were considered significantly associated with booster dose acceptance. Model fitness was checked by the Hosmer and Lemeshow goodness-of-model fit test. Odds ratios and 95 % confidence intervals were used to assess the statistical significance between dependent variable and independent predictor variables. No missing data was recorded in this analysis.

## 3 Results

### 3.1 Participant's socio-demographic and socio-economic profiles

In total, data from 607 potentially eligible people were included in this analysis. The investigator evaluated the inclusion criteria for each participant who provided informed consent and anonymous data on COVID-19 VBD willingness. However, a significant portion of rural people may felt anxiety to attend an in-person quantitative interview session; thus, skepticism from the participation occurred in some cases. Face-to-face investigation process blocked the import of missing data in the analysis.

The following Table 1 displays the respondent's demographic profiles. According to the demographic overview, a majority (33.3 %) of the participants were 26–33 years aged, and more than half (59.5 %) portions of the participant were male. Among the participants, 22.6 % were housekeepers, and the highest 21.6 % had Honours or equivalent education as a tertiary degree. We have collected the data from all divisional areas of Bangladesh, of which the highest 21.1 % were recruited from the Khulna region. Most of the participants (78 %) were Muslim by religion, and 26.5 % of the participants were contaminated by coronavirus. In total, 64.3 % people completed two doses primer COVID-19 vaccine, while most of the individuals (74 %) reported no severe symptoms after administering it.

**Table 1:** Overview of the respondent's demographic profile (N = 607).

Variables	N	%
Age distribution		
18–25 years	142	23.4
26–33 years	202	33.3
34–41years	107	17.6
42–49 years	59	9.7
50–57 years	23	3.8
58–65 years	46	7.6
65+ years	28	4.6
**Gender**		
Male	361	59.5
Female	246	40.5
**Educational status**		
Illiterate	113	18.6
Primary	97	16.0
High school or equivalent	64	10.5
HSC or equivalent	105	17.3
Honors or equivalent	131	21.6
Diploma	48	8.0
Masters or above	49	8.1
**Religion**		
Muslim	473	78.0
Hindu	98	16.1
Others	36	6.0
**Marital status**		
Single	207	34.1
Married	389	64.0
Others	11	1.8
**Occupation**		
Teacher	13	2.1
Government employee	63	10.4
Private employee	99	16.3
Small business	112	18.6
Housekeeper	137	22.6
Farmer and day-laborer	131	21.6
Health worker	17	2.8
Students	35	5.8
**Geographical location**		
Rajshahi	116	19.1
Dhaka	84	13.8
Chattogram	54	8.9
Khulna	128	21.1
Barishal	62	10.2
Sylhet	56	9.2
Rangpur	79	13.1
Mymensingh	28	4.6
**Area of residence**		
Urban	228	37.6
Rural	379	62.4
**Experience of COVID-19 positive**		
Yes	161	26.5
No	446	73.5
**Vaccination status**		
First dose	217	35.7
Completed two doses	390	64.3
**Side effects symptoms (after primer doses)**		
No symptom	449	74.0
Mild to moderate symptom	131	21.6
Severe symptom	27	4.4
**Intention to receive booster doses**		
Yes	425	70.0
Unsure	62	10.2
No	120	19.8

### 3.2 Results of the questionnaire

Table 2 shows the descriptive statistics of the instrument (predictor variables and response variable) used in the study. “COVID-19 VBD acceptance/willingness” was the outcome event of this study.

**Table 2:** Descriptive results of the questionnaire.

Operational definition	Yes response AF (RF)	Mean	SD
I would like to accept booster vaccine anytime (Yes = 1, otherwise = 0)	425 (70.0 %)	0.70	0.459
I confirm that booster doses are similarly safe as were regular two doses (Yes = 1, otherwise = 0)	419 (69.03 %)	0.74	0.450
I think the vaccine booster dose is very efficacious (Yes = 1, otherwise = 0)	488 (80.4 %)	0.70	0.457
I am worried about post-vaccination side effects of booster vaccine dose (Yes = 1, otherwise = 0)	506 (83.4 %)	0.62	0.486
Vaccine booster doses may provide repeated immunity to body (Yes = 1, otherwise = 0)	411 (67.7 %)	0.50	0.500
I am well-communicated about booster vaccine dose (Yes = 1, otherwise = 0)	527 (86.8 %)	0.62	0.486
I have trust on booster vaccination (Yes = 1, otherwise = 0)	397 (65.4 %)	0.71	0.456
Therapeutic benefit of booster doses overweigh the perceived risk (Yes = 1, otherwise = 0)	401 (66.06 %)	0.52	0.500
I have adequate information on vaccine booster doses (Yes = 1, otherwise = 0)	523 (86.2 %)	0.71	0.456
Booster vaccination is essential to protect my community people (Yes = 1, otherwise = 0)	402 (66.2 %)	0.64	0.481
I do not need booster doses because I strictly follow the community measures (Yes = 1, otherwise = 0)	176 (29.0 %)	0.31	0.464
Booster vaccine doses should be mandated for people (Yes = 1, otherwise = 0)	156 (25.7 %)	0.47	0.500
Booster vaccine doses prevent the arrival of new corona variant (Yes = 1, otherwise = 0)	297 (32.5 %)	0.65	0.486
Booster vaccine provides long-lasting immune responses (Yes = 1, otherwise = 0)	384 (63.3 %)	0.49	0.500
Receiving a vaccine dose is very usual to me (Yes = 1, otherwise = 0)	543 (89.5 %0	0.59	0.496

### 3.3 COVID-19 vaccine booster dose acceptability and the potential factors

The pooled COVID-19 vaccine booster dose acceptance rate was 70.0 % (95 % CI: 67─73) among the general people in Bangladesh.

Table 3 represents the findings of the binary regression analysis. The analysis revealed that, out of 14 potential factors, “efficacy”, “repeated immunity”, “communication”, and “trust” showed highly significant positive association (aOR = 2.151 95 % CI: 1.391─ 3.508, aOR = 2.033 95 % CI: 1.299─ 3.181, and aOR = 2.552 95 % CI: 1.557─4.183 respectively; *p=*0.002, *p=*0.002, *p=*0.000, and *p=*0.000, respectively) and “equal safety”, “risk-benefit ratio” and “community protection” had significant positive association (aOR = 1.739 95 % CI: 1.070─2.825, aOR = 1.712 95 % CI: 1.116─2.627, and aOR = 1.628 95 % CI: 1.395─0.998; *p* = 0.026, *p* = 0.014, and *p* = 0.049, respectively) with vaccine booster dose acceptance*.* However, fear of post-vaccination “side effects” showed highly significant negative (aOR = 0.393 95 % CI: 0.237─0.674, *p* = 0.000) associations with COVID-19 VBD acceptance among the Bangladeshi public. Peoples who believed that COVID-19 VBD has severe side-effects were less likely interested to uptake it.

**Table 3:** Binary logistic regression analysis *(B= Beta coefficient, S.E= Standard Error, Wald = Wald statistics, AOR (95 % CI) = Adjusted Odds Ratio (95 % Confidence Interval)*.

Variable	B	S.E.	Wald	Sig.	Exp(B)	95 % C.I. for EXP(B)	
						Lower	Upper
Equal safety*	0.553	0.248	4.989	0.026	1.739	1.070	2.825
Efficacy**	0.766	0.250	9.411	0.002	2.151	1.391	3.508
Side effects**	−0.933	0.254	13.479	0.000	0.393	0.239	0.674
Repeated immunity**	0.710	0.228	9.651	0.002	2.033	1.299	3.181
Communication**	0.952	0.231	16.982	0.000	2.592	1.648	4.078
Trust**	0.937	0.252	13.818	0.000	2.552	1.557	4.183
Risk-benefit ratio*	0.538	0.219	6.053	0.014	1.712	1.116	2.627
Information sufficiency	−0.266	0.252	1.111	0.292	0.766	0467	1.257
Community protection*	0.465	0.236	3.871	0.049	1.628	1.395	1.998
Preventive measures	0.148	0.237	0.392	0.513	1.160	0.729	1.844
Booster mandate	0.329	0.232	2.015	0.156	1.398	0.882	2.187
Variants control	0.234	0.512	4423	0.131	1.421	0.943	1.984
Long-lasting immunity	0.401	0.244	2.689	0.101	1.493	0.925	2.409
Culture	0.025	0.242	0.011	0.918	1.025	0.638	1.649
Constant	−1.075	0.437	6.050	0.014	0.341		

note: ** = significant at <0.01, * = significant at <0.05, level of significance. The result of Cox & Snell R square test in binary model showed that, the outcome variable was 28.8 %─40.8 % explained by the joint function of predictive variables in the study which was satisfactory and assumed to be good level. The significance level for Omnibus tests of model coefficients was found significant (p < 0.05) while it was insignificant (p = 0.901) for Hosmer and Lemeshow test. A larger p-value of Hosmer and Lemeshow's goodness-of-fit test indicates indicative of the goodness of fit of the model.

### 3.4 Pearson's chi-square test

Table 4 displays the Pearson's chi-squared test results and odds ratio. Females were slightly more trend to uptake a booster shot (odds = 1.26), however, the effect was insignificant (*p>0.05)* found in chi-squared values. Therefore, the general population of Bangladesh showed no statistically significant risk of booster vaccination hesitant in any group (gender).Table 4: Pearson's chi-square test and risk estimation.

**Table 4:** Pearson's chi-square test and risk estimation.

Chi-Square tests								
		Value	Asymptotic significance (2-sided)			Exact sig. (2-sided)		Exact sig. (1-sided)
Pearson Chi-square		1.666^a^	0.197					
Continuity correction^b^		1.441	0.230					
Likelihood ratio		1.658	0.198					
Fisher's exact test						0.206		0.115
Linear-by-linear association		1.664	.197					
N of valid cases		607						
Risk estimate				Value	95 % Confidence Interval			
					Lower		Upper	
	Odds ratio for gender: (Female/Male)			1.261	0.887		1.793	
	For cohort I intend to accept vaccination anytime = No			1.175	0.921		1.499	
	For cohort I intend to accept vaccination anytime = Yes			0.932	0.836		1.039	
	N of valid cases			607				

## 4 Discussion

The COVID-19 vaccine became clinically approved in the middle of 2021and proven proved to be an effective preventive medicine in reducing coronavirus infection, hospitalizations, and unusual deaths. This study investigated COVID-19 booster vaccine acceptance intent and explored the key antecedents of VBD acceptance and hesitancy among the general people in Bangladesh. Since vaccines are basic medical interventions to stop the pandemic, significant logistic supports allowed the global researchers and scientists to include many vaccines into the platform with various technologies. In addition to developing and providing vaccines, a key aspect is people's willingness to be vaccinated [51,52]. Vaccine hesitancy may result in some people accepting some but not all recommended boosters or postponing or delaying the schedule.

Findings from this study showed that, the pooled booster vaccine acceptance rate was 70.01 % among the Bangladeshi people. Most of the studies conducted on primer vaccination in Bangladesh reported that 60–80 % of people had positive attitudes to accept a COVID-19 vaccine [[39], [40], [41], [42], [43]]. Worldwide, numerous studies have investigated varied COVID-19 VBD acceptance across different sub-population groups, including 44.6 % university community in Jordan [26], 75.3 % university people in Italy [27], 58.28 % adult people in India [30], 78.66 % health professionals in Nepal [32], 87.8 % university community in Germany [45]^,^ 74.5 % students and health professionals in Poland [46], 72.7 % university community in Belgium [47], 62 % adult in the USA [48], 87.6 % general people in Malaysia [53]^,^ 93.7 % Chinese general people [54], 71 % adult poles in Poland [55], 76.8 % older citizen in China [56], and 71.1 % health care workers in Saudi Arabia [57], 77.76 % general people in Pakistan [58] and 73.8 % health personnel in Singapore [59] declared their willingness to boost 3rd dose vaccine against the COVID-19 pandemic. These findings are supportive to our findings.

In recent years, a number of studies have shown that several multi-dimensional factors played important roles in making vaccination decisions among large population groups with substantial geographical variability [[60], [61], [62]]. Factors such as safety and efficacy regarding the COVID-19 vaccine were among the factors affecting people's attitudes toward receiving the booster vaccine. , The results are consistent with those of previous studies [4,7,58,61,63]. The post-vaccination side effect was identified as a primary reason of booster dose skepticism in many Asian countries [[63], [64], [65]] as well as other region [55]. Roy et al., deduced that the side effect issue has led to fear and the formation of attitudes about receiving periodic COVID-19 vaccines among rural people in Bangladesh [42]. Hence, the post-vaccination side effects has been shown to be associated negatively with VBD acceptance in our research. According to our study's results trust, communication, and culture were important predictors of making booster dose decisions among the general people in Bangladesh. There was a critical relationship between the level of trust in vaccination and confidence regarding vaccine safety, side effects, and efficacy [48,66]. In contrast, public corruption is one of the reasons for cross-country variation in vaccination progress [67], and misinformation about COVID-19 vaccines led to public distrust of vaccine uptake [68]. The use of credible and culturally competent health communication influences positive health behaviors, guides decision-making, addresses concerns, and builds trust. Furthermore, tailored and transparent health communication with remote people is crucial for minimizing misinformation and vaccine unwillingness [69,70].

Individual decision to uptake a COVID-19 VBD was influenced by several multi-dimensional characteristics [71], in which VBD acceptance rate was directly proportional to the level of public trust [72]. Previous research evident that, several predictors including trust, communication, and information were the most common concerns of COVID-19 vaccine decisions among the general public [73] and the peoples of educational sectors [74,75] in Bangladesh. In our study, community protection and repeated immunity were recognized as significant predictors of booster acceptance. Due to the high level of accountability that exists in social and community care, community health protection has been identified in our study as a key factor of VBD acceptance. In the recent past same predictors including protection of family health, community health, and individual health were recognized as various altruistic promoters of COVID-19 VBD in many countries [45,46]. These results are in agreement with previously published studies from different countries.

To ensure the study's external validity, we have incorporated a large data sample. The variability in respondents' socio-demographic attributes and the high sample size gives strength to provide the study's generalizability, ensuring COVID-19 VBD confidence and willingness among the public. The ability to prevent severe illness, community infection, and symptomatic infection was associated with increased antibody and booster intake, respectively [76]. COVID-19 booster doses have the potential against infection-related consequences, thus limiting new variant arrival; however, the continued need for non-pharmaceutical intervention after booster vaccination is also recommended for the people [77]. Since good governance could play a critical role in the race of national immunization, considerable efforts should be provided by the WHO and wealthier countries for ensuring equal distribution of COVID-19 vaccines to the countries of less functional governance [78].

### 4.1 Practical implications

A major benefit of this study comes from its ability to provide evidence-based booster dose promotional planning to health policymakers, stakeholders, and vaccine promoters. A rigorous health intervention involving key messages delivered by community leaders and vaccine policymakers would provide insight into factors contributing to booster vaccine acceptance and hesitancy. Consequently, this study's findings may contribute to overcoming barriers and propagating facilitators for a nationwide booster vaccine roll-out while improving public engagement with vaccination programs; this may assist governments when designing a booster dose protocol. The results of this study could provide scientific evidence for further observational studies of COVID-19 VBD consequences by further investigating the nexus between booster reluctance and associated confounding variables of interest. Since periodic COVID-19 vaccine acceptance rate was found to be increased over time [79] and the pattern of COVID-19 vaccine hesitancy can be modified [80], this study largely contributes to the design of a long-term surveillance study for monitoring the temporal changes in factors associated with COVID-19 booster vaccination decisions.

### 4.2 Strengths and limitations

This is the first study focused on COVID-19 booster vaccine acceptability among Bangladeshi people, invited people via face-to-face interviews, and used a novel tool to identify the key determinants of VBD acceptance. Our study's strength comes from the fact that we thoroughly analyzed the data and that it included participants in every age group between 18 and 65. Since repeated immunity and community health protection led to booster dose receptivity, hence, the prospective health benefits over risk have been established. The identification of trust and communication as a positive predictor for booster acceptance added a new value to the existing field of research. This study addressed the following limitations: the cross-sectional nature of this study limited the ability to validate the causal relationship between the variables we investigated. As a second issue, our sample size was insufficient when compared with the population of Bangladesh as a whole. Therefore, it is imperative to take caution when generalizing the study results to other areas of Bangladesh and overseas. In addition, public vaccination behavior and decision can be altered with frequent changes in perceived health risks, disease exposure, vaccine characteristics, and vaccine availability. However, some other confounding factors may also cause vaccine hesitancy, some of which were not explicitly defined in this study. Finally, sequential alteration and temporal changes in factors associated with COVID-19 booster vaccine reluctance may occur over time and with local and regional context.

## 5 Conclusions

Booster vaccination is necessary to guarantee the persistent herd immunity against current COVID-19 and to lessen breakthrough infection of the newly arrived variant. This study concluded that, COVID-19 VBD acceptance was found moderately high among the general people of Bangladesh and numerous multi-dimensional factors were associated with VBD acceptance. This study confirmed a positive attitude towards the COVID-19 VBD was an outcome of this study, regardless of the circumstances, as far as safety, efficacy, perceived benefits, communication, trust, and community resistance are concerned. Post-vaccination side effects fear was the reason for booster dose skepticism as well as a barrier to administering COVID-19 vaccine third shots. Effective communication and educational interventions are the best preferred approaches to make vaccine's data more available publicly.

## Funding

This research did not receive any specific support from funding agencies in the public, commercial, or not-for-profit sector.

## Ethics declarations

The Ethical review committee **approved the study as exempt and the project was registered into the** official logbook (Ref. No.-JUST/PHAR/161007/2022).

## Consent to participate

Informed consent was obtained from the participants. This study did not harm the individual, and the individual was free to reject participation.

## Data availability statement

Data associated with our study been deposited into a following publicly available repository:

Figshare: “Data set_COVID-19 booster vaccine_ Bangladesh” https://doi.org/10.6084/m9.figshare.24279157.

## CRediT authorship contribution statement

**Debendra Nath Roy:** Writing – review & editing, Writing – original draft, Supervision, Software, Resources, Methodology, Investigation, Formal analysis, Data curation, Conceptualization. **Shaheb Ali:** Writing – original draft, Software, Resources, Formal analysis, Data curation, Conceptualization. **Ashish Kumar Sarker:** Writing – original draft, Software, Resources, Formal analysis, Data curation. **Ekramul Islam:** Writing – review & editing, Visualization, Validation, Supervision, Project administration, Investigation, Conceptualization. **Md Shah Azam:** Writing – review & editing, Visualization, Validation, Supervision, Project administration, Methodology, Investigation.

## Declaration of competing interest

The authors declare that they have no known competing financial interests or personal relationships that could have appeared to influence the work reported in this paper.
