# Supplementary material for: Acceptance of COVID-19 vaccine booster dose among the people of Bangladesh: A cross-sectional study
Source: Heliyon. 2023 Nov 11;9(11):e22215. doi: 10.1016/j.heliyon.2023.e22215 (PMC10694156; doi:10.1016/j.heliyon.2023.e22215)
Supplement: Multimedia component 1 [file mmc1.pdf]

Questionnaire

Dear Participants,

We are conducting a research project entitled as **"Acceptance of COVID-19 vaccine booster dose (VBD) from Bangladeshi people"**. Your answer will be used for academic purpose only. All of your given information is strictly confidential.

I would very much appreciate if you answer all of the questions carefully.

Sincerely yours

Participant: I clearly understand the research objectives and willing to provide anonymous data ☐Yes

1. Your age:☐ 18-25 Years; ☐ 26–33 Years; ☐ 34 -41 Years; ☐ 42-49 Years; ☐50-57 Years; ☐58-65 Years;☐ 65+ Years
2. Educational status: ☐ Illiterate ☐Primary ☐Secondary or equivalent ☐ Higher Secondary or equivalent ☐ Honors or equivalent ☐ Diploma☐ Masters or above
3. Gender: ☐Male ☐ Female ☐ Others

4. Religion: ☐ Muslim; ☐ Hindu;☐ Christian; ☐ Buddhist

5. Occupation: ☐ Teacher; ☐ Government employee ☐ Private employee; ☐ Small business; ☐ Housekeeper; ☐ Farmer and day-laborer; ☐ Health worker; ☐ Students ☐Others

6. Marital status:☐ Single ☐ Married ☐Others

7. You are from which Division: ☐ Rajshahi; ☐ Dhaka; ☐ Chattogram; ☐ Khulna; ☐ Barishal; ☐ Sylhet; ☐ Rangpur; ☐ Mymensingh

8. Area of residence: ☐ Urban ☐Rural

9. Presence of co-morbidity: ☐Yes ☐ No

10. Experience of COVID positive ☐Yes ☐ No

11. Present vaccination status: ☐ received first dose ☐ completed two doses

12. Level of side effects found after receiving primary vaccine dose: ☐ No symptom ☐ Mild to moderate ☐ Severe

13. Are you willing to uptake COVID-19 vaccine booster dose, when it is available? ☐ Yes ☐Unsure ☐No

---

14. I confirm that booster doses are similarly safe as were regular two doses : ☐ Yes ☐Unsure ☐ No

15. I think the vaccine booster dose is very efficacious: ☐ Yes ☐Unsure ☐ No

16. I am worried about post-vaccination side effect of booster vaccine dose: ☐ Yes ☐Unsure ☐ No

17. Vaccine booster doses may provide repeated immunity to body: ☐ Yes ☐Unsure ☐ No

18. I am well-communicated about booster vaccine dose: ☐ Yes ☐Unsure ☐ No

19. I have a trust on booster vaccination: ☐ Yes ☐Unsure ☐ No

20. Therapeutic benefit of booster doses overweigh the perceived risk: ☐ Yes ☐Unsure ☐ No

21. I have adequate information on vaccine booster doses: ☐ Yes ☐Unsure ☐ No

22. Booster vaccination is essential to protect my community people: ☐ Yes ☐Unsure ☐ No

23. I do not need booster doses because I strictly follow the community measures: ☐ Yes ☐Unsure ☐ No

24. Booster vaccine doses should be mandated for people: ☐ Yes ☐Unsure ☐ No

25. Booster vaccine doses prevent the arrival of new corona variant: ☐ Yes ☐Unsure ☐ No

26. Booster vaccine provides long-lasting immune responses: ☐ Yes ☐Unsure ☐ No

27. Receiving a vaccine dose is very usual to me: ☐ Yes ☐Unsure ☐ No

Thank you very much for your co-operation
