# Supplementary material for: Acceptance of COVID-19 vaccine booster dose among the people of Bangladesh: A cross-sectional study
Source: Heliyon. 2023 Nov 11;9(11):e22215. doi: 10.1016/j.heliyon.2023.e22215 (PMC10694156; doi:10.1016/j.heliyon.2023.e22215)
Supplement: Multimedia component 2 [file mmc2.pdf]

ফার্মেসী বিভাগ
যশোর বিজ্ঞান ও প্রযুক্তি বিশ্ববিদ্যালয়
যশোর - ৭৪০৮, বাংলাদেশ
যোগাযোগ : +৮৮০১৭০৯-৮১৮১১৬, +৮৮০২৪২১৪২০৩৪
ই-মেইল: chairman.phar@just.edu.bd

Department of Pharmacy
Jashore University of Science and Technology
Jashore-7408, Bangladesh
Contact Number : +8801709-818116, +880242142034
E-mail: chairman.phar@just.edu.bd

Ref. No.-JUST/PHAR/23/964

Date: 25/05/2023

This is to inform that, the research project emitted as "Acceptance of COVID-19 vaccines booster dose among the people of Bangladesh" has been supervised by Debendra Nath Roy, Assistant Professor, Department of Pharmacy, Jashore University of Science and Technology, Jashore, Bangladesh. Since data were collected anonymously and no clinical intervention was applied to the encounters, so the protocol was ethically approved as exempt. The project was registered into the Department's logbook (Ref. No.-JUST/PHAR/161007/2022) as well.

Please, don't hesitate to contact me regarding this issue.

Thanking you,

Dr. Kishor Mazumder
Chairman
Department of Pharmacy
Jashore University of Science & Technology
Jashore-7408, Bangladesh.

Dr. Kishor Mazumder
PhD (Osaka, Japan), Postdoc (CSU, Australia)
Associate Professor and Chairman
Department of Pharmacy
Jashore University of Science and Technology (JUST) &
Adjunct Sr. Lecturer
School of Optometry and Vision Science UNSW Medicine
University of New South Wales (UNSW) Sydney, NSW, Australia
